# Complete genome of *Erwinia spp. str*. LJJL01 isolated from waste charcoal

**DOI:** 10.1128/mra.00347-25

**Published:** 2025-08-15

**Authors:** Lakshika Dissanayake, Jeffrey G. Linger, Lahiru N. Jayakody

**Affiliations:** 1 School of Biological Science, Southern Illinois University Carbondale 2254, Carbondale, Illinois, USA; 2 National Renewable Energy Laboratory 53405, Golden, Colorado, USA; 3 Fermentation Science Institute, Southern Illinois University Carbondale 2254, Carbondale, Illinois, USA; California State University San Marcos, San Marcos, California, USA

**Keywords:** *Erwinia spp. str*. LJJL01, Oxford Nanopore sequencing, synthetic microbiology

## Abstract

We present the complete genome sequence of *Erwinia spp. str*. LJJL01, isolated from waste charcoal in Colorado, USA, using Oxford Nanopore sequencing. This sequence provides important insights into this bacterium’s metabolic and catabolic robustness to utilize sugars, acids, and aromatics, highlighting its potential as a bio-industrial strain for various feedstocks.

## ANNOUNCEMENT

**Fig 1:**
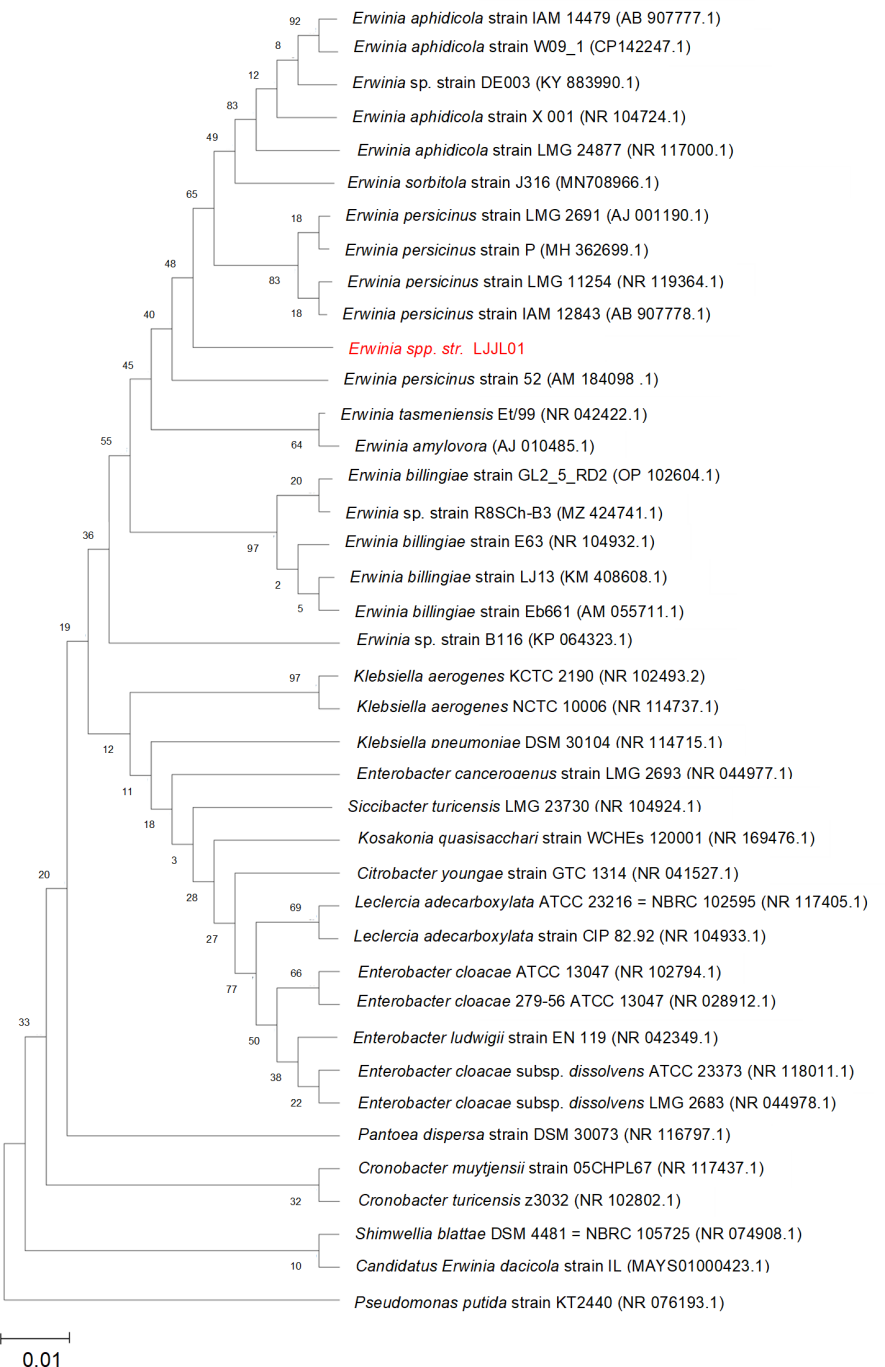
Maximum likelihood phylogenetic tree based on 16S rRNA sequences of 35 species aligned with the test strain. Full-length 16S rRNA genes aligned by SILVA (version 1.2.11) and unrooted maximum likelihood phylogenetic tree was generated by MEGA X, with 1,000 bootstraps. The scale bar represents 1% identity dissimilarity between sequences.

*Erwinia spp. str*. LJJL01 was isolated from the top 6 inches of a soil sample that contains hardwood lump charcoal in 2016 from Denver, Colorado, USA (Global positioning system coordinates: 39.778447–105.036688)*,* based on its ability to grow on an agar plate containing minimal media (M9) supplemented with 5 mM of lignin-derived monomers such as syringol or catechol (1). Earlier this strain was identified as *Erwinia aphidicola* LJJL01 based on a partial sequence of 16S rRNA. The full-length 16S rRNA gene sequence based on the assembled genome reported here and phylogenetic analysis suggests that this bacterium is another species of *Erwinia* and is designated as *Erwinia spp. str*. LJJL01 (Fig. 1) (2, 3). The strain was deposited in the U.S. Department of Agriculture’s Agricultural Research Service Culture Collection under the number NRRL B-65680. The first draft genome sequence of *Erwinia spp. str*. LJJL01 was completed by the Joint Genome Institute in 2018 (ID- 2751185488) using PacBio RS II, sequencing with 122.0× genome coverage and assembly with HGAP (version 2.3.0_p5) and annotated using IMG Annotation Pipeline (version 4.15.1; NCBI GenBank ID: SNXL01000001:SNXL01000004) but was not described in peer-reviewed literature.

Here, we present the re-sequenced and closed genome of *Erwinia spp. str*. LJJL01 using nanopore sequencing. A single colony of *Erwinia spp. str*. LJJL01 from a Luria-Bertani agar plate was used to inoculate 5 mL of M9-glucose medium, incubated overnight at 30°C and 225 rpm to obtain cell pellets for genomic DNA extraction. DNA was extracted using GeneJET genomic DNA purification kit following the manufacturer’s instructions (Thermo Fisher Scientific, Waltham, MA, USA). DNA concentration was measured using nanoquant plate in TECAN Sunrise Infinite plate reader (TECAN, Switzerland). Genome sequencing was performed using Oxford Nanopore Technology (ONT) at Eurofins Genomics LLC (Louisville, KY, USA). DNA was prepared for sequencing using the Rapid Barcoding Kit 96 V14 SQK-RBK114.96 following the manufacturer’s instructions (ONTs, UK). Mechanical shearing or size selection of the library was not adopted in the analysis. Sequencing was performed using Oxford Nanopore GridION loaded with V14 MinION flow cell (Oxford Nanopore Technologies, UK). The base calling was carried out using Dorado (version 4.3) (4). The resulting reads undergo quality filtering using NanoPack2 software, are assembled with Flye assembler (version 2.9.2), polished with Pilon (version 1.24), and the assembly quality is assessed using QUAST (version 5.2.0). The default parameters were used for the analysis (5–7). A summary of the results is provided in Table 1. Additionally, the assembly is annotated using the NCBI Prokaryotic Annotation Pipeline (version 6.7) and available under GCA_047300805.1 (8–10).

**TABLE 1:** Nanopore sequencing statistics and genomic features of *Erwinia spp. str*. LJJL01^*a*^

Overall assembly	
Total assembly length (bp)	4,951,323
No. of contigs	4
Largest contig	4,751,215
GC %	56.56
Total reads	145,853
Total bases (Mb)	464,063,254
Average coverage depth	94
N50	4,751,215
L50	1
Assembly completeness	
Marker lineage	Enterobacteriaceae (UID5054)
Completeness	99.13%
Contamination	0.20%
Species determination	
Best species match	*Candidatus Erwinia dacicola* strain IL
Sequence identity to best species match	92.5557%
Summary of annotations	
tRNAs	83
rRNAs	22
ncRNAs	10
CDSs	4,510
Pseudogenes	110

^*a*^ CDS, Coding DNA Sequence; GC, guanine and cytosine; ncRNAs, Non-coding RNA.

Nanopore genome sequencing of *Erwinia spp. str*. LJJL01 revealed the presence of four contigs, including the genome of size 4,751,215 bp, and three native plasmids of sizes 104,322 bp, 89,813 bp, and 5973 bp. Of note, chromosomes and plasmids were confirmed as circular using Flye (6). The genome includes genes that encode enzymes for metabolic pathways involving sugars (glucose, xylose, arabinose, galactose, and maltose), acids (lactic and acetic), and aromatics (such as catechol, vanillin, and benzoate).

## Data Availability

The complete genome for *Erwinia spp. str.* LJJL01 has been deposited in NCBI under BioProject PRJNA1208068, including the ONT base-called FASTQ file, SRR32982199. The annotated genome (FASTA) is listed under the NCBI accession number CP178576.1. Native plasmids are available under accession numbers CP178574.1, CP178575.1, and CP192608.1.

## AFTER EPUB

*[This article was published on 15 August 2025 with an error in the author byline. The byline was corrected in the current version, posted on 21 August 2025.]*
